# Progress in Ophthalmology

**Published:** 1901-12-07

**Authors:** 


### PROGRESS IN OPHTHALMOLOGY.

**Treatment of "After Cataract."**[1]—At the recent annual meeting of the British Medical Association a discussion took place as to the treatment of "after cataract," which practically means the remaining opaque lens capsule. Many opinions were given as to methods of "needling" and the needles themselves. These instruments, although sharpened at the ends on both edges for the purpose of cutting rather than tearing the membrane, generally end by tearing and not cutting. This manœuvre, of course, causes a good deal of dragging of the iris and ciliary processes, especially if the membrane is a thick and tough one and does not yield fairly readily. Many operators advocated cutting through the membrane with a ground-down Graefe knife, which is the ordinary instrument used in making the corneal section in extraction of cataract. We were rather surprised at the respect, not to say awe, with which many surgeons approached this operation, no less a person than Mr. Power giving it as his opinion "that at least a year should elapse before any secondary operation was undertaken, and then only with hesitation and doubt, and that in most cases if the vision were moderately good the patient should be contented, and not run any further risks." For our own part we have seen very few troubles following the operation: once a glaucoma was set up and had to have sclerotomy done for its relief, and once or twice some slight iritis has followed, but nothing more than this and nothing approaching the disastrous failures referred to by Mr. Power.

**The Workmen's Compensation Act and the Testing of Workmen's Sight.**[2]—This very pertinent question was also brought up at the same meeting, by the reading of a paper on the above subject by Mr. H. E. Jones. He says:—"Under the Workmen's Compensation Act of 1897, a person whose wage-earning power has been lessened by any injury incurred while at work, is entitled to receive from his employer half the amount per week by which his average weekly wage is reduced, or an equivalent capital sum. In estimating the amount of compensation due for any injury, everything turns upon the answer to the question—To what extent, if any, has the injury reduced the person's wage-earning power?" It has been our lot to have to examine several men for injury to their sight on behalf of insurance companies. These companies insure the employer against compensation, and have no knowledge of the particular men, till the employer applies to the company, through the employee, to carry out their share of the bargain, so they have no means of testing the workman's sight. In two cases, lately, men came having injured one eye, and we found that the other eye was an old amblyopic one, and in no way the worse for the accident; but still the workman was out of employment; and the insurance company had to compensate him for this loss. Mr. Jones suggests that all workmen should be tested before they are taken on, and that the result of this test should be recorded against their names on the wages sheet. He suggests that the test should be of "the rough and ready type," such as dot counting at 5 or 6 feet, which could be made by a clerk or the manager, and which would suffice to bring out most defects, including errors of refraction; it must be a test of the vision of the naked eye, and, above all, the eyes must be tested separately. He foresees three objections to this:—First, it is held by some ophthalmologists that it is always possible to tell by objective examination whether or not there existed any defect in either or both eyes before the alleged accident. This objection he very properly does not admit, as he says, it is a matter of common experience that intelligent persons may have a serious defect in one eye without finding it out, and after an injury to such an eye the patient is often prepared to swear, in good faith, that the eye was perfectly normal before the accident. Secondly, it may be objected on behalf of the men that the inquiry into the condition of their sight might lead to the dismissal or degradation of a large number of men. This objection he answers by saying It is not a likely occurrence. But suppose such a result did follow the application of eye tests—it would probably benefit the men in the long run, as those who were unfit for the work they were doing, would find work they could do, before their defective sight had caused injury to themselves or their fellow-workmen. Moreover, a higher standard of sight required would entail compensation for injuries which are now considered comparatively trivial, such as a partial loss of sight in one eye. Third, it may be objected that, as eye injuries form only a small part of injuries for which compensation is claimed (one in 20), why examine the eye, and not make a general examination. To this he answers that any general examination would have to be made by a medical man, and would entail a good deal of expense, but the dot test that he has proposed for the eyes could be done by any intelligent clerk and would cost nothing. We have personally spoken to several insurance managers in this strain, but they still say "it is impossible to do anything, and they must just take the risk."

[1] Brit. Med. Jour., Nov. 2, 1892. [2] Brit. Med. Jour., Nov. 2, 1892.

